# Application and practice of trimodal prehabilitation model in preoperative management of patients with lung cancer undergoing video-assisted thoracoscopic surgery

**DOI:** 10.3389/fsurg.2022.1047977

**Published:** 2023-01-06

**Authors:** Liping Yao, Hui Chen, Bei Xue

**Affiliations:** Department of Nursing, Shanghai Chest Hospital, School of Medicine, Shanghai Jiao Tong University, Shanghai, China

**Keywords:** lung cancer, trimodal prehabilitation, rapid rehabilitation surgery, functional capabilities, prognosis

## Abstract

Lung cancer is one of the malignant tumors with high mortality worldwide. To date, the most effective treatment of non—small cell lung cancer (NSCLC) is still surgical resection. Video-assisted thoracoscopic surgery has become the main surgical approach. Tumor patients are the high-risk perioperative population. At present, how to optimize perioperative management measures to improve the patient's body function and promote the rehabilitation after video-assisted thoracoscopic surgery is a hot research topic for medical staff. In this study, 148 patients with lung cancer were selected as the research object, to analyze and discuss the application value of trimodal prehabilitation model in preoperative management of patients with lung cancer undergoing video-assisted thoracoscopic surgery.

## Introduction

Lung cancer has become one of the common malignant tumors in clinic, and the incidence rate of lung cancer is increasing year by year (1). Video-assisted thoracoscopic surgery (VATs) has become the main surgical approach for chest diseases due to its advantages of less trauma and rapid recovery of patients. The latest guidelines issued by the National Comprehensive Cancer Network (NCCN) in 2018 proposed that video-assisted thoracoscopic surgery should be preferred for patients with NSCLC (2). However, due to impaired lung function, obesity, and weakness, some patients with lung cancer have poor body reserve function, which cannot ensure the efficacy of the operation and increases the risk of postoperative complications (3). Therefore, it is urgent to find effective methods to fight for more patients' operation opportunities, improve the operation efficacy, reduce postoperative complications and improve the prognosis in clinical practice.

The study found that compared with the traditional rehabilitation measures which only carry out postoperative rehabilitation, maintaining a good physiological and psychological state before surgery is more conducive to increasing the reserve function and making the body in a better functional state (4, 5). Pre-rehabilitation is an emerging preoperative management strategy based on the enhanced recovery aftersurgery (ERAS). The new preoperative optimization strategy proposed in this scheme mainly includes the trimodal prehabilitation measures of exercise, nutritional support and psychological intervention in the preoperative stage, aiming to improve the body function of patients to withstand the stress of surgery, accelerate the rehabilitation process and improve the clinical outcome of patients (6). As a new pre-operative intervention mode, trimodal prehabilitation is being accepted by scholars both in China and abroad, and has been verified by many studies that pre-operative intervention in surgical patients can promote their rehabilitation (7). However, it still lacks its application value in thoracoscopic surgery for lung cancer in China. This study analyzed the application of trimodal prehabilitation model in preoperative management of patients with lung cancer undergoing video-assisted thoracoscopic surgery. The report is as follows.

## Information and methods

### General information

A total of 160 patients who received video-assisted thoracoscopic surgery for lung cancer in the inpatient department of Shanghai Chest Hospital from June 2021 to December 2021 were selected. According to the inclusion and exclusion criteria, 148 patients were finally taken as research subjects and randomly divided into an intervention group and a control group, with 74 cases in each group. All patients signed informed consent forms, and this study has been approved by the Medical Ethics Committee of our hospital.

### Inclusion criteria

① According to the 2020 NCCN Guidelines for the Diagnosis and Treatment of Non-small Cell Lung Cancer and the eighth edition of international lung cancer pathological staging criteria, all patients were stage I-II lung cancer patients with feasible surgical resection; ② Patients who underwent video-assisted thoracoscopic surgery; ③ The patient has no physical activity disorder, is conscious, and can understand and cooperate with medical staff; ④ Age ≤75 years old.

### Exclusion criteria

① Patients with lung cancer in which the tumor has invaded the peripheral organs and extensive adhesion to the pleura; ② Patients with a previous history of ipsilateral pulmonary surgery; ③ Lung tumors cannot undergo one-lung ventilation; ④ Patients with severe complications before operation, including patients with severe hematological and immune system diseases; ⑤ Patients with cardiac function ≥Class ≥III; ⑥ Patients with compact adhesion of thoracic cavity explored during operation and tumor invading thoracic wall; ⑦ Patients who switched from video-assisted thoracoscopic surgery to thoracotomy due to massive hemorrhage; ⑧ Patients who underwent pneumonectomy by changing the operation mode during the operation; ⑨ Pet-name ruby postoperative patients with active bleeding tendency; ⑩ Patients with incomplete or untrue clinical data were excluded.

### Research methods

Patients in the control group received routine perioperative intervention. They completed medical history inquiry, laboratory and physical examination before hospital admission and assessment of general preoperative conditions. Routine nursing measures and health guidance were given during the perioperative period, including hospital admission propaganda and education, diet guidance, respiratory function exercise, preoperative patient preparation and postoperative precautions.

The intervention group was given trimodal prehabilitation intervention strategy on the basis of the control group. ① Firstly, a triple pre-rehabilitation group is set up, with the head nurse of our department as the team leader, and the team members included two experienced nursing supervisors and several responsible nurses. Under the group leader's organization, all team members were trained in the pre-rehabilitation nursing practice program, and after confirming that all ward nurses had mastered the program operation process, the intervention group patients were given nursing measures. ② On the day of hospitalization for patients with pre rehabilitation guidance, distribution of pre rehabilitation guidance manual, and guidance will be recorded daily activities, guidance form including oral, manual and demonstration. ③ After the operation, the physical condition of the patient was evaluated, and the physician wrote the exercise prescription and gave the patient exercise support. Ways of exercise: a. aerobic exercise: you can arrange exercises according to your actual situation. the common exercise ways are floor training, fast walking and jogging. Stair-climbing training: Climbing 45 steps at the normal stair-climbing speed constituted one group of sports, with two to three groups in succession. 20 min each time, 2 times/day. Brisk walking or jogging can also be performed. The duration of exercise can be initially set at 10–20 min, and finally increased to 30 min, 2 times/day. It was advisable for patients to exercise without increasing obvious fatigue, and when the degree of self-perceived fatigue was heavy, the exercise intensity could be reduced. The target heart rate was calculated based on the patient's age, i.e., target heart rate = (220−age) × (70%–80%). b. Respiratory trainer Deep breathing training: 10–15 min/time, 3 times/day. c. Abdominal breathing exercise: The patient was guided to the abdominal breathing method at 10–15 min/time, 3 times/day. ④ Nutrition support by combined medical care and establishment of ward nutrition intervention group composed of competent doctor, responsible nurse and dietician. The NRS 2000 scale was used to assess the nutritional status of patients. The patients with a score of <3 points ate a balanced diet, and the patients with a score of ≥3 points were malnourished. After evaluation, intervention was carried out, and the group made dietary plan according to the patient's situation. 20 g whey protein was added into the beverage 1 h after exercise. ⑤ Professional scale was used to evaluate the psychological state of patients and psychological consolation was carried out accordingly. At the same time, to guide patients to carry out relaxation training, the specific content is as follows. Patients were required to empty their stools and wear loose clothes before training. Keep the room quiet, neat, the light is appropriate. Instruct patients to close their eyes and concentrate in the most comfortable position. Breathe naturally, and slowly clenched his fist when inhaling to feel the muscle tension (about 5–10 s), and then slowly loosen your fist when exhaling to feel the muscle relaxation (about 10–30 s). The feelings of muscle groups of arm, head and face, neck, shoulder, chest, abdomen, back, hip, lower limbs and both feet were felt orderly until the muscles of the whole body were relaxed, which lasted for about 20 min. At the same time, take listening to music to relax training. Keep the indoor environment quiet and comfortable, and use headphones. Wearing headphones, the music volume should be kept under 60 dB, and instrumental music such as Chinese classical folk music and world famous music will be played. The intervention of the two groups was ended after the first week of operation.

### Observation indicators

(1) Activity capacity: The results of the 6-minute walking test (6 MWT) before intervention, 1 d before operation and 30 days after operation in the two groups were compared. The patients of the two groups walked in a straight line in a corridor with a length of 30 m for 6 min, and the total walking distance of the patients was finally measured and recorded as 6 MWT (8). During testing, put three chairs in the corridor for rest. During the test, attention was paid to the presence of warning signs of fall. During the test, the patient's oxygen saturation, heart rate and Brog score were continuously monitored. Once the patient developed dyspnea, chest pain and chest tightness, and progressive decline in oxygen saturation, the test was immediately stopped. Experimental preparation of epinephrine and nitroglycerin tablets and other rescue medication, abnormal timely notify the doctor.
(2) Psychological status: The results of Hospital Anxiety and Depression Scale (HADS) before intervention, 1 d before operation and 30 days after operation between the two groups were compared. HADS consisted of anxiety (anxiety, a) and depression (depression, d) sub-scales, with 7 questions for anxiety and depression respectively, which were rated by the patients themselves with a total score of 21 points. 0–7 points indicate no anxiety and depression symptoms, 8–10 points indicate possible anxiety and depression, and 11–21 points indicate affirmation of anxiety and depression (9).
(3) Nutrition status: Elbow venous blood was collected before intervention, 1 day before operation and 30 days after operation, and once before the patient was discharged. All automated hematology analyzer was used to test the nutritional indicators of the patients. Serum albumin (albumi, ALB) was detected by bromocresol green method, prealbumin (PA) was detected by rate nephelometry, and transferrin (TRF) level was detected by nephelometry.
(4) Comparison of the incidence of postoperative complications and the postoperative hospital stay between the two groups.
(5) Evaluate the patient's nursing satisfaction at discharge. According to the specific situation of our department, a satisfaction questionnaire was designed to obtain the satisfaction data of patients' families from the monthly satisfaction survey conducted by our department. The content of the satisfaction survey included safety, environment, comfort, accessibility, respect for patients, nursing technology, health education, communication and so on. The full score of the satisfaction questionnaire was 100. The higher the score was, the higher the patients' satisfaction with the nursing care would be.

### Data statistics

SPSS Statistics 22.0 software was used for statistical analysis. Measurement data were expressed as $(\bar{x}\pm S)$. Paired-samples *t*–test was used for intra-group comparison before and after treatment, and *t*-test was used for inter-group comparison. The count data were expressed as *n* (%) using the *χ^2^* test. For inter-group comparison at multiple time points, the overall difference was analyzed using the repeated measures analysis of variance. *P* < 0.05 was considered as the difference with statistical significance.

## Result

### Comparison of general information

The general data of the two groups were not statistically significant (*P* > 0.05), and they were comparable, as shown in Table 1.

**Table 1:** Comparison of two groups of general data.

Group	Age (years old)	Gender (*n*)		Surgical approach (*n*)			ASA grading (*n*)	
		Male	Female	Pulmonary lobe	Lung segment	Wedge	I	II
Intervention group (*n* = 74)	53.59 ± 5.74	43	31	59	6	9	46	28
Control group (*n* = 74)	54.18 ± 5.66	40	34	62	4	8	43	31
*t/χ* ^2^	0.630	0.247		0.533			0.254	
*P*	0.530	0.619		0.766			0.615	

### Comparison of activity abilities of patients between the two groups before and after intervention

**Figure 1:**
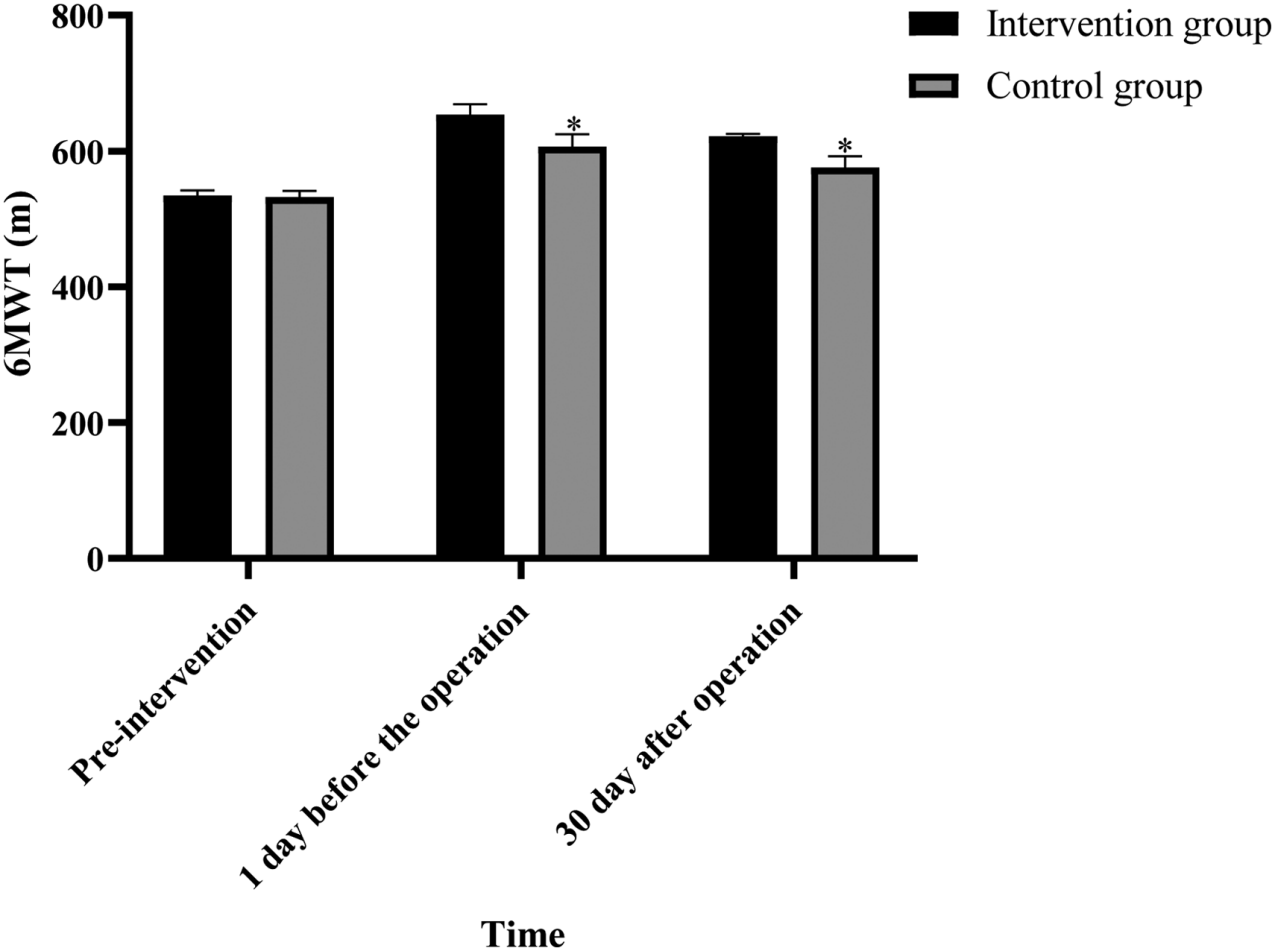
Comparison of 6 MWT between the two groups at different times (Note: compared with the intervention group, *P < 0.05).

There was no significant difference in 6 MWT between the two groups before intervention (*P* > 0.05). One day before surgery, the 6 MWT in both groups was higher than that before intervention and higher in the intervention group than in the control group. On the 30th day after surgery, the 6 MWT values in the two groups were lower than that one day before surgery, but it was higher in the intervention group than in the control group (*P* < 0.05), as shown in Figure 1.

### Comparison of psychological states of patients between the two groups before and after intervention

**Figure 2:**
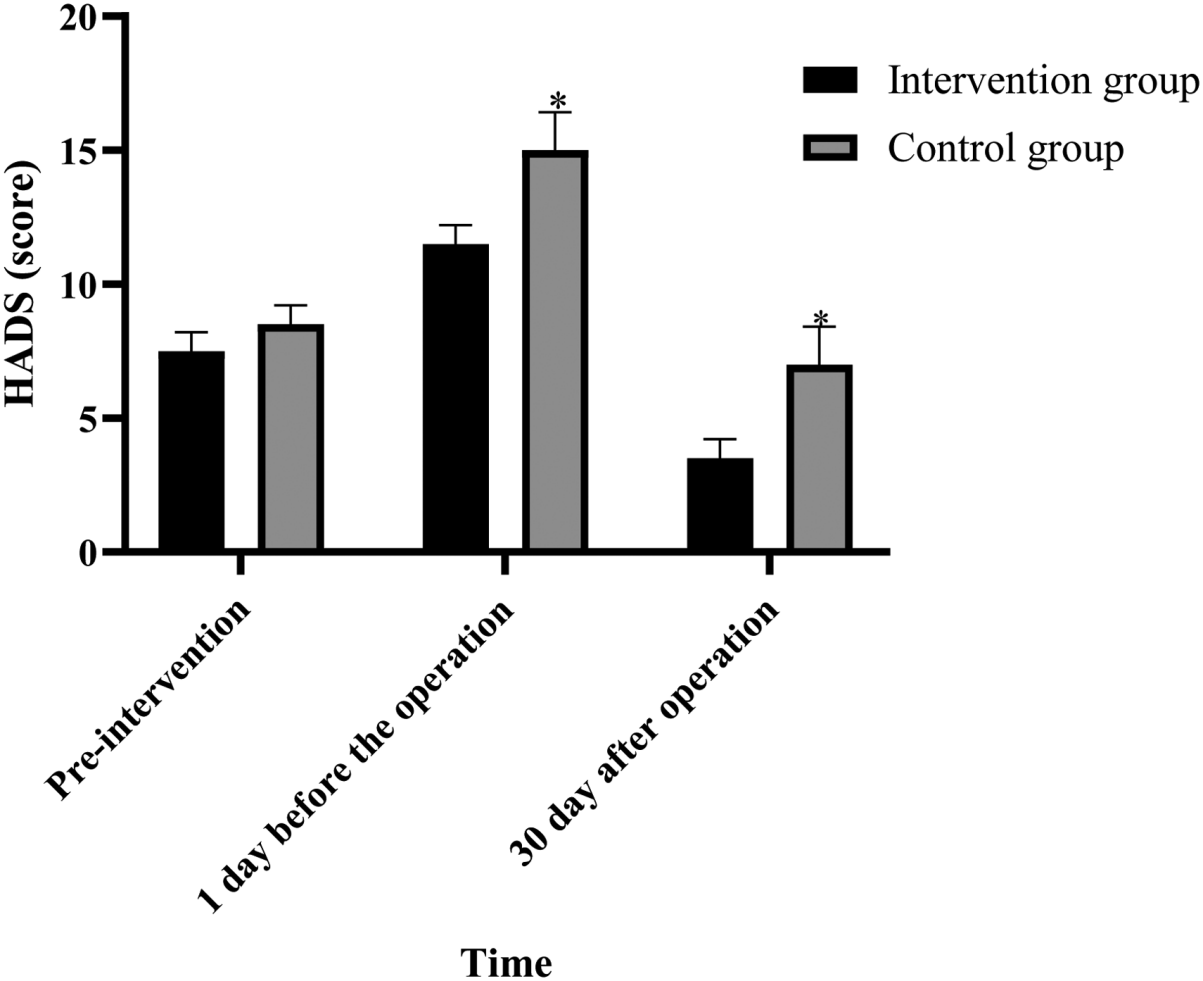
Comparison of HADS scores in two groups at different times (Note: compared with the intervention group, *P < 0.05).

There was no significant difference in HADS scores between the two groups before intervention (*P* > 0.05). One day before surgery, the scores of the two groups were higher than those before intervention, but the scores in the intervention group were lower than those in the control group. The HADS scores of the two groups were lower than those of the one-day intervention group and the control group 30 days after the operation (*P *< 0.05), as shown in Figure 2.

### Comparison of ALB, PA and TRF levels between the two groups before and after intervention

**Figure 3:**
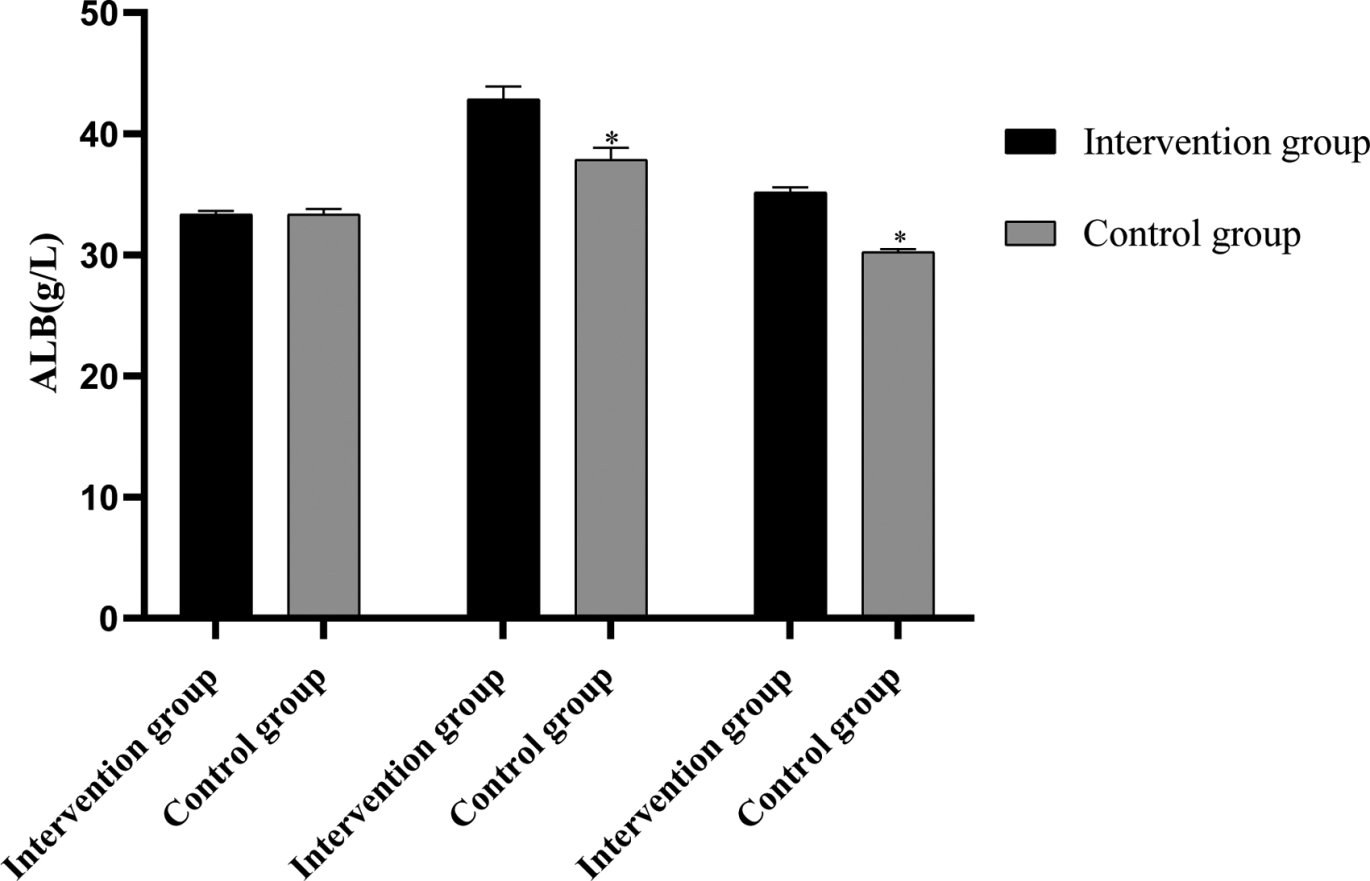
Comparison of ALB levels between the two groups before and after intervention (Note: compared with the intervention group, *P < 0.05).

**Figure 4:**
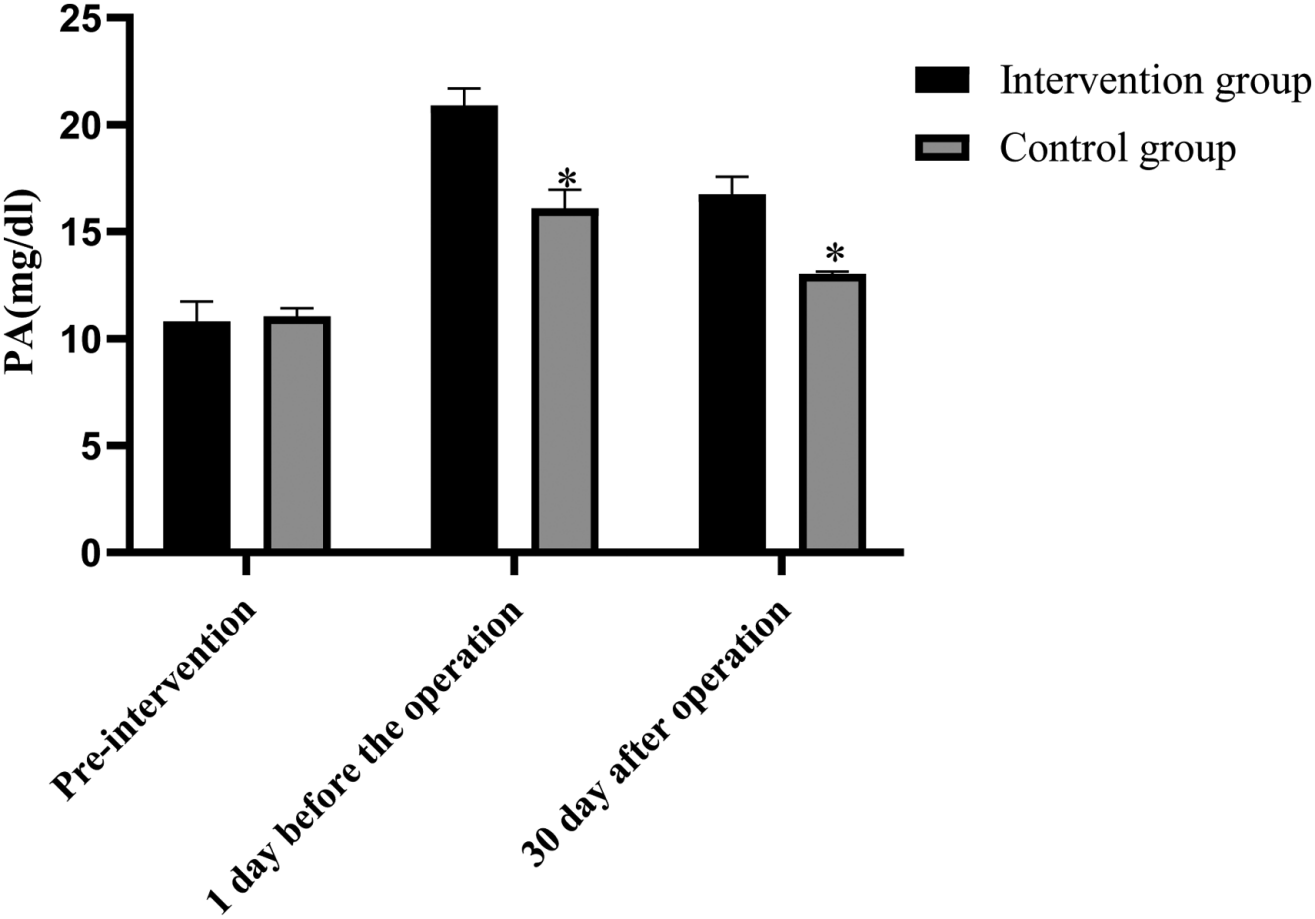
Comparison of PA levels between the two groups before and after intervention (Note: compared with the intervention group, *P < 0.05).

**Figure 5:**
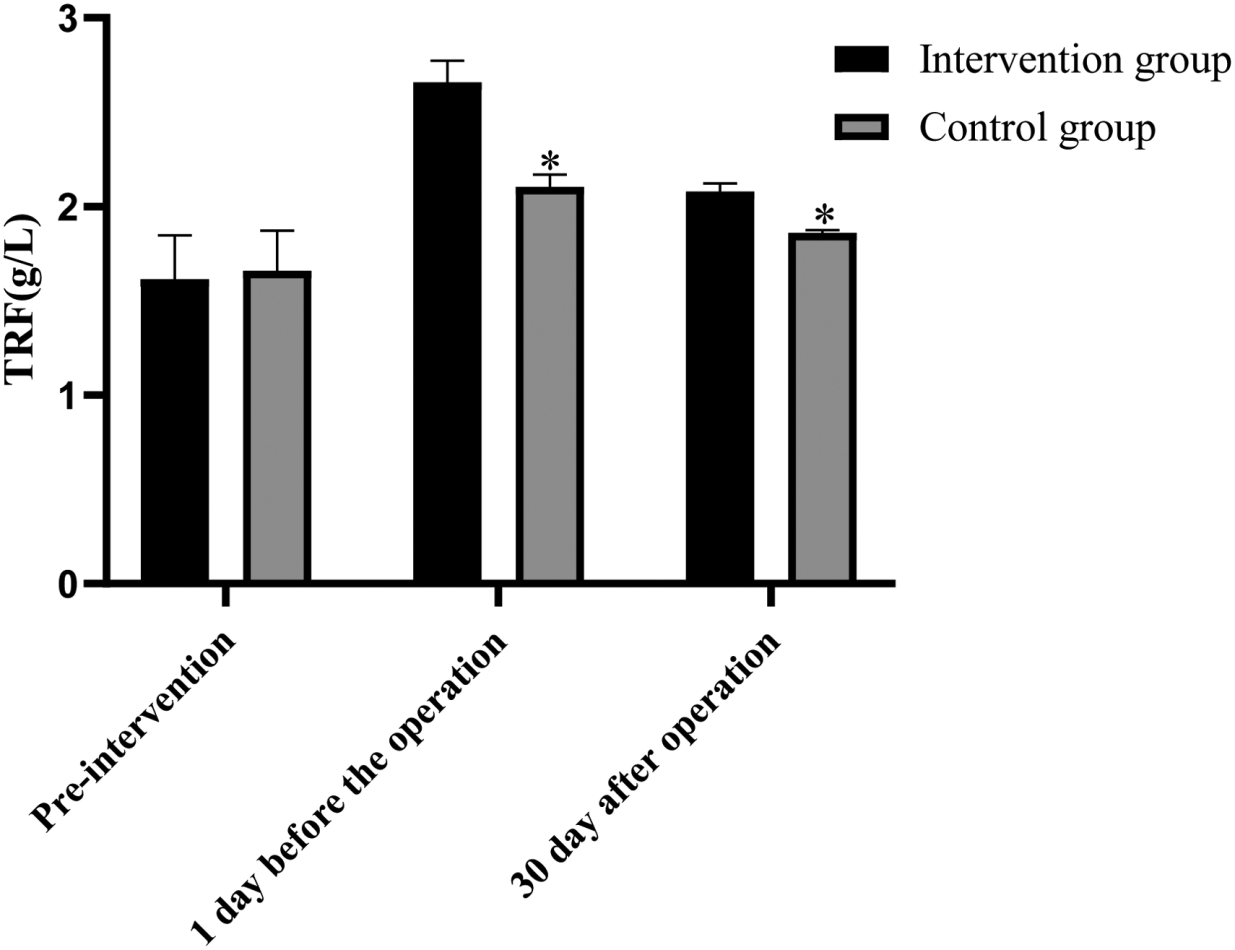
Comparison of TRF levels between the two groups before and after intervention (Note: compared with the intervention group, *P < 0.05).

Before intervention, the levels of ALB, PA and TRF in the two groups were not statistically significant (*P *> 0.05). One day before surgery, the levels of ALB, PA and TRF in the intervention group were higher than those before intervention (*P* < 0.05), but the levels of ALB, PA and TRF in the control group were not statistically significant as compared with those before intervention (*P* > 0.05). On the 30th day after surgery, the levels of ALB, PA and TRF in the intervention group were lower than those on the 1st day before surgery (*P* < 0.05), but their levels were not statistically significant as compared with those before intervention (*P *> 0.05). The levels of ALB, PA and TRF in the control group were lower than those 1 day before surgery and before intervention (*P *< 0.05). The levels of ALB, PA and TRF in the intervention group were higher than those in the control group one day before surgery and 30 days after surgery (*P* < 0.05), as shown in Figures 3–5.

### Comparison of the incidence of postoperative complications and postoperative hospital stay between the two groups before and after intervention

The incidence of complications in the intervention group was lower than that in the control group, and the postoperative hospital stay was shorter than that in the control group (*P* < 0.05), as shown in Table 2.

**Table 2:** Comparison of postoperative complications and hospitalization duration between the two groups before and after intervention.

Group	Postoperative complications						Postoperative hospitalization time (d)
	Atelectasis	Empyema	Bronchopleural fistula	Subcutaneous emphysema	Pulmonary infarction	Total	
Intervention group (*n* = 74)	1 (1.35)	0 (0.00)	0 (0.00)	1 (1.35)	0 (0.00)	2 (2.70)	7.16 ± 1.55
Control group (*n* = 74)	3 (4.05)	1 (1.35)	1 (1.35)	2 (2.70)	1 (1.35)	8 (10.81)	9.57 ± 1.27
*t/χ* ^2^						3.861	10.346
*P*						0.049	<0.001

### Comparison of nursing satisfaction between the two groups

The nursing satisfaction degree in the intervention group was higher than that in the control group (*P *< 0.05), as shown in Table 3.

**Table 3:** Comparison of nursing satisfaction between the two groups ($\bar{x}\pm S$, score).

Group	Nursing satisfaction
Intervention group (*n* = 74)	13.95 ± 3.14
Control group (*n* = 74)	13.18 ± 3.16
*t*	1.175
*P*	0.243

## Discussion

Studies have shown that the incidence of postoperative complications in lung cancer patients undergoing minimally invasive surgery is still as high as 26.2%–36.3% (10, 11). The core of ERAS concept is to reduce the trauma and stress on patients in each stage of the perioperative period, in order to shorten the treatment time, reduce complications and reduce the mortality rate (12). In recent years, the concept of ERAS has become a consensus in the field of surgery. However, previous efforts of ERAS to improve the prognosis of patients have mainly focused on the intraoperative (laparoscopic surgery, epidural anesthesia, etc.) and postoperative (analgesia, early feeding, rehabilitation activities, etc.), without preoperative intervention (13). The trimodal prehabilitation strategy is a new concept of preoperative management proposed based on ERAS (14).

There is increasing evidence that objective assessment of physical fitness and nutritional status can predict postoperative recovery. This study showed that after intervention, the motor function, psychological state and nutritional state of patients in the two groups were significantly improved compared with those before intervention. However, the levels of MWT, ALB, PA and TRF in the intervention group were higher than those in the control group at 6 MWT one day before surgery and 30 days after surgery, and the postoperative hospital stay in the intervention group was shorter than that in the control group. This indicates that the trimodal prehabilitation model has a significant effect on improving the physiological and psychological state of patients with lung cancer undergoing video-assisted thoracoscopic surgery, and can shorten the postoperative treatment time.

To further analyze the cause value of triple pre-rehabilitation model in thoracoscopic surgery for lung cancer. On the one hand, the first step of the triple pre-rehabilitation model is to establish a triple pre-rehabilitation model team, which can help to the functional state of the patient at a high level throughout the surgery and recover to the preoperative baseline level more quickly after surgery by developing a reasonable optimization plan in accordance with the specific situation of the patient before the surgery (15). On the other hand, different from the control group that only carried out health education, the intervention group that received triple pre-rehabilitation mode was based on exercise training, and at the same time carried out nutritional support and psychological intervention measures, which can adjust the preoperative functional status of patients with lung cancer undergoing video-assisted thoracoscopic surgery from different aspects (16). Among them, aerobic exercise is the main form of exercise training, which includes two complementary exercise types of endurance and strength training. Moreover, there are many methods of aerobic exercise in the intervention group, including climbing training, brisk walking, jogging, etc., to avoid the decrease of compliance caused by boring training, which is helpful to enhance the aerobic metabolic capacity and muscle strength of patients, enhance their physiological reserves, and facilitate postoperative rehabilitation (17). In addition to exercise, respiratory exercise is also an important component of exercise support for patients undergoing video-assisted thoracoscopic surgery for lung cancer (18). Low respiratory muscle strength is one of the risk factors of pulmonary complications after thoracoscopic surgery for lung cancer. Respiratory exercise can strengthen the strength of respiratory muscles, facilitate the discharge of sputum, increase vital capacity and prevent the incidence of postoperative pulmonary complications such as atelectasis during perioperative period. In addition, preoperative nutritional support is also one of the key measures for pre-rehabilitation (19). Different degrees of malnutrition are common in patients with lung cancer, and the patients are in stress state of high catabolism after surgery, which further aggravates the malnutrition of the body and seriously affects the postoperative rehabilitation. Therefore, nutritional support for patients undergoing video-assisted thoracoscopic surgery for lung cancer can increase their preoperative nutritional reserve,simultaneously provide the basis for exercise training and postoperative nutritional consumption, and reduce the incidence of postoperative infection.

In addition, studies have reported that anxiety and depression significantly inhibit immune function and can affect postoperative wound healing, leading to adverse outcomes (20). Therefore, psychological intervention for lung cancer patients undergoing thoracoscopic surgery is very important to reduce stress response and enhance the effect of pre-rehabilitation. In this study, the perioperative anxiety and depression were effectively relieved by individualized explanation of perioperative plan and related knowledge, as well as psychological intervention, relaxation training and respiratory training. In the trimodal prehabilitation model, sports and psychological support are mutually reinforcing. Physical exercise accompanied by sympathetic excitation, blood flow and oxygen consumption increased, resulting in changes in excitatory neurotransmitters, good stimulation of the central nervous system, can make people feel better, from which to obtain a sense of exercise pleasure, satisfaction and self-confidence, but also conducive to the improvement of anxiety and depression before surgery (21). In this study, the HADS scores of the intervention group were lower than those of the control group 1 day and 30 days after operation (*P* < 0.05), further verifying that the application of the triple pre-rehabilitation mode in the pre-operative management of lung cancer patients undergoing video-assisted thoracoscopic surgery can improve the psychological state of patients and facilitate the postoperative recovery.

Notably, some important issues were also found in the application of the triple pre-rehabilitation model. Patients undergoing video-assisted thoracoscopic surgery for lung cancer may have a number of pre-operative conditions that hinder exercise planning, including anxiety, depression, malnutrition, comorbidities, and the tumor itself, all of which can affect their compliance with treatment and intervention. Therefore, before the trimodal prehabilitation, adequate education and education on the content of pre-rehabilitation are needed to encourage patients to timely adjust their mentality before surgery and strictly follow the strategy of the triple pre-rehabilitation model. At the same time, when carrying out sports training, it is necessary to avoid excessive exercise intensity, so as to avoid fatigue, injury or poor compliance. For continuous improvement of physical strength, the intensity of exercise should be slowly and gradually increased. In addition, it is necessary to maintain the diversity of plans and adjust the physical and mental state of patients from multiple aspects. In addition, the influence of no difference in complications between the two groups on the postoperative treatment time of the two groups needs to be confirmed by a larger sample size.

In summary, the application of trimodal prehabilitation model in preoperative management of patients with lung cancer undergoing video-assisted thoracoscopic surgery is conducive to improving the functional state and psychological state of patients, preventing complications, and improving nursing satisfaction.

## Data Availability

The original contributions presented in the study are included in the article/Supplementary Material, further inquiries can be directed to the corresponding author/s.
